# Low-volume cycling training improves body composition and functionality in older people with multimorbidity: a randomized controlled trial

**DOI:** 10.1038/s41598-021-92716-9

**Published:** 2021-06-28

**Authors:** Eduardo Carballeira, Karla C. Censi, Ana Maseda, Rocío López-López, Laura Lorenzo-López, José C. Millán-Calenti

**Affiliations:** Gerontology and Geriatrics Research Group, Instituto de Investigación Biomédica de A Coruña (INIBIC), Complexo Hospitalario Universitario de A Coruña (CHUAC), SERGAS, Universidade da Coruña, 15071 A Coruña, Spain

**Keywords:** Geriatrics, Diseases

## Abstract

Physical exercise, when practiced regularly and in adequate doses, is a proven nonpharmacological measure that helps to prevent and reverse noncommunicable diseases, as well as reduce mortality rates from any cause. In general, older adults perform insufficient physical activity and do not meet the doses recommended by the World Health Organization for the improvement of health through physical activity. However, there is little evidence on adequate doses of exercise in older people, especially in those with multimorbidity. Our main aim was to evaluate the effect of a 6-week intervention on health-related outcomes (body composition, hemodynamic and functionality changes) in 24 individuals aged 65 and older with multimorbidity in a randomized controlled trial. The intervention consisted of a very low volume (60 min per week) of low-to-moderate intensity exercise training (perception of effort from 3 to 6 on an 11-point scale). After the intervention, blood pressure was significantly (*p* = 0.038) reduced in the exercise group (EG), with a higher reduction in men. Furthermore, the EG decreased their waist circumference (*p* = 0.005), a proxy of abdominal adiposity, and demonstrated an increased likelihood (73%) that a randomly selected change in muscle mass score from the EG would be greater than a randomly selected change score from the control group. The exercise intervention was particularly effective in enhancing the functionality of older adults with multimorbidity, especially in walking speed and balance skills. Perceptually regulated intensity during exercise training seemed to be a very interesting strategy to train individuals with low physical fitness and comorbidities. This study is registered with Clinicaltrials.gov (NCT 04842396).

## Introduction

Aging has been recognized as a risk factor for most chronic diseases, and the presence of more than two diseases (i.e., multimorbidity), which is frequent in almost two out of three older adults, has been related to an increased risk of disability and frailty, a decrease in quality of life, and mortality^1^. Physical activity (PA) has been suggested as a “polypill”, which, when practiced regularly, acts as a nonpharmacological intervention that generates an adaptive response that has been shown to prevent and treat noncommunicable diseases (NCDs) and up to 35 chronic conditions^2,3^. Regular physical activity (rPA) reduces rates of all-cause mortality, compresses morbidity (i.e., reduces the length of time a person spends sick or disabled), decreases healthcare costs, and has relatively minimal adverse effects compared to drugs^4^. Moreover, rPA provides immune competency across the lifespan, helping, among other factors, in the prevention of transmissible diseases such as viruses and bacterial infections, and it has been suggested that this may even limit and delay the aging of the immune system^5^. There is also increasing evidence from previous research that rPA, especially cardiorespiratory-type exercises, improves mental health and delays cognitive impairment in older adults^6,7^.

It has been estimated that 27.5% of the world’s population in 2016 did not meet the recommendations established for the member states of the World Health Organization (WHO) for health-enhancing physical activity^8^. Furthermore, physical inactivity increases with age; healthy older adults and those with disabilities and chronic diseases often have less access to safe, accessible, affordable, and appropriate spaces and places in which they can be physically active^9^. Recommendations of rPA in adults aged 65 years and older state a minimum of 150 min of moderate-intensity aerobic physical activity throughout the week, at least 75 min of vigorous-intensity aerobic physical activity throughout the week, or an equivalent combination of moderate- and vigorous-intensity activity^10^. The limitation of the aforementioned WHO recommendation lies in its underlying scientific evidence, which is mainly based on studies with healthy older adults and may not be applicable in older people who are habitually characterized by low physical fitness and/or NCDs^11,12^. These recommendations, which are also the basis of the physical activity promotion policies in some health national departments, have been criticized for the possible discouraging effect that they can cause by posing a barrier for inactive individuals to be "activated"^12–14^. The increase in physical activity (a behavior) and the consequent increase in physical fitness (an attained state) must be the main message that must reach the entire population, especially those with sedentary behavior (activities <1.5 metabolic equivalents or METs) and with very low physical fitness^12^. Older individuals tend to be in this situation, especially those who are institutionalized, with rates of the prevalence of frailty in nursing homes higher than 60%^15^, and approximately 40% were still prefrail^16^. Lower doses of exercise intensity and volume, i.e., below those recommended by the WHO, have been demonstrated to improve physical fitness and other health-related markers (body composition, functional status, glucose homeostasis, bone health, psychological well-being, and overall quality of life) in adult populations of all ages^17,18^. Furthermore, a recent systematic review of reviews and meta-analyses^6^ showed that moderate-intensity physical activity may be sufficient for reducing the risk of all-cause dementia and that some of the protective benefits of physical activity for older adults are accrued well below current guidelines for health. In summary, it seems indispensable to study adequate doses of exercise for older people who often have low levels of physical activity and fitness, who spend a large amount of time sitting down, and whose multimorbidity keeps them away from exercising.

One of the main problems when prescribing exercise doses in older adults is setting an intensity of exercise and maintaining it throughout the training session. Monitoring the internal load during a training session is a challenging issue since a determined external load (i.e., gait velocity, cycle power) prescribed for a group of older people with a high prevalence of multimorbidity is not a "one size fits all" exercise. The common method employed in monitoring the internal load when exercising in cardiorespiratory exercise blocks is heart rate (HR) registration; however, older adults with multimorbidity, who often consume medications, compromise the reliability of HR for monitoring exercise response^19^. In contrast, the rating of perception of effort (i.e., RPE) has been used as an effective tool to regulate exercise intensity^20^, even in people receiving β-blockade therapy^21^ and those with mild cognitive impairment^22^. However, to our knowledge, the effect of perceptually guided training in older adults with multimorbidity has not been studied. Therefore, the present work aimed to study the effects of perception-regulated low-volume and low-to-moderate intensity training on body composition, hemodynamic parameters, and functional performance in older adults with multimorbidity.

## Results

The baseline characteristics of the participants can be found in Table 1. The control and exercise groups were homogeneous in all variables.

**Table 1:** Demographic and health characteristics of the participants.

	Exercise group (n = 12)	Control group (n = 12)	*p*
**Demographics**			
Age (years; mean ± SD)^a^	80.4 ± 7.8	82.1 ± 7.6	0.602
Female [n (%)]^b^	7 (58.3%)	8 (66.6%)	0.673
Weight (kg; mean ± SD)^a^	69.5 ± 12.3	68.2 ± 12.1	0.792
Height (cm; mean ± SD)^a^	160.0 ± 9.0	154.0 ± 10.0	0.104
BMI (kg/m^2^; mean ± SD)^a^	26.6 ± 3.8	28.6 ± 5.5	0.311
**Type of institutionalization (n (%))**			
Nursing home (NH)^b^	8 (66.7%)	8 (66.7%)	1.000
Day center (DC)^b^	4 (33.3%)	4 (33.3%)	1.000
**Number of patients using a walking aid (n (%))**^b^	6 (50.0%)	8 (66.6%)	0.132
**Comorbidity conditions**			
GDS score [n (%)]^b^			0.881
GDS 2	4 (33.3%)	4 (33.3%)	
GDS 3	5 (41.7%)	4 (33.3%)	
GDS 4	3 (25.0%)	4 (33.3%)	
CCIa [median (IQR)]^c^	4.5 (4–7)	4.0 (4–5.75)	0.458
Myocardial infarct [n (%)]	2 (16.7%)	0 (0.0%)	
Congestive heart failure [NYHA I y II; n (%)]	3 (25.0%)	1 (8.3%)	
Peripheral vascular disease [n (%)]	1 (8.3%)	1 (8.3%)	
Cerebrovascular disease [n (%)]	2 (16.7%)	2 (16.7%)	
Mild dementia [n (%)]	3 (25.0%)	4 (33.3%)	
Chronic pulmonary disease [n (%)]	0 (0.0%)	1 (8.3%)	
Diabetes [n (%)]	3 (25.0%)	2 (16.7%)	
Moderate or severe renal disease [n (%)]	3 (25.0%)	2 (16.7%)	
Any tumor [n (%)]	2 (16.7%)	0 (0.0%)	
Hypertension [n (%)]	6 (50.0%)	8 (66.7%)	
**Medication**			
Total number (mean ± SD)^a^	9.8 ± 3.7	9.0 ± 2.9	0.546
Participants taking BPM [n (%)]^b^	8 (66.7%)	9 (75.0%)	0.408
Participants taking B-blockers [n (%)]^b^	4 (33.3%)	1 (8.3%)	0.132

*BMI* body mass index, *BPM* medications that lower or potentially lower blood pressure, *CCIa* Charlson comorbidity index adjusted by age, *GDS* global deterioration scale, *IQR* interquartile range.

^a^*t*-test, ^b^Chi-squared test, ^c^Mann–Whitney *U* test.

### Body composition changes

Data on changes in body composition are presented in Table 2. There was a significant interaction of time x group in weight (*t*_20_ = 3.01, *p* = 0.007) and waist circumference (WC) (*t*_22_ = −3.14, *p* = 0.005), revealing that participants in the experimental group (EG) moderately increased their weight (with stochastic superiority of the interaction of the time (post- vs. preintervention) and the group [EG vs. control group (CG)] (AEG-CG) = 73%) and largely decreased their WC (AEG-CG = 17%). However, the adjusted post hoc comparison showed significant changes in EG only for WC (*t*_22_ = 3.47, *p* = 0.013). Participants from the EG reduced 4.6 cm (95% confidence interval (CI) [−7.4; −1.7] cm, AEG-CG = 17%, large) of WC compared with CG as an effect of the intervention. Overall, there were no detectable changes in muscle mass or fat mass as an effect of the intervention. Notwithstanding these findings, the likelihood that a randomly selected change on the EG muscle mass score would be greater than that on the CG muscle mass score was 73%. When adjusted by sex, there was an interaction (time × group × sex) for the weight (β = 1.98, 95% CI [0.31; 3.66], *t*_20_ = 2.32, *p* = 0.031) and no significant increase in muscle mass (β = 1.00, 95% CI [−0.05; 2.06], *t*_18.96_ = 1.86, *p* = 0.079). The simple effects analysis indicated that males in the EG significantly increased their weight (β = 1.28, 95% CI [0.34; 2.22], *t*_20_ = 2.83, *p* = 0.010) and barely increased their muscle mass (β = 0.58, 95% CI [−0.01; 1.17], *t*_19.95_ = 2.06, *p* = 0.053).

**Table 2:** Changes in body composition of exercise (EG) and control (CG) groups.

	EG		Post- versus preintervention		CG		Post- versus preintervention		Interaction of time × group	
	Pre-	Post-	MD [95% CI]	*p* value	Pre-	Post-	MD [95% CI]	*p* value	B estimate [95% CI]	*p* value
			Cohen's dz^a^ [95% CI]				Cohen's dz^a^ [95% CI]		AEG-CG	
Weight, kg	70.8 ± 11.6	71.7 ± 11.6	0.86 [0.28; 1.44]	0.054	68.6 ± 12.2	68.2 ± 12.2	− 0.43 [− 1.04; 0.18]	0.890	1.29 [0.45; 2.13]	0.007**
			0.71 [0.08; 1.35]				− 0.23 [− 0.80; 0.35]		73%	
			Medium				Small		Large	
WC, cm	99.8 ± 12.4	96.2 ± 12.4	− 3.6 [− 5.60; − 1,60]	0.013*	101.3 ± 12.4	102.3 ± 12.4	1.0 [− 1.0; 3.0]	1.000	− 4.6 [− 7.40; − 1.70]	0.005**
			− 0.96 [− 1.64; − 0.27]				0.30 [− 0.28; 0.87]		17%	
			Large				Small		Large	
MM, kg	24.4 ± 3.4	24.7 ± 3.4	0.29 [− 0.07; 0.65]	0.530	22.1 ± 3.6	21.9 ± 3.6	− 0.24 [− 0.63; 0.15]	0.530	0.53 [0.7e^−3^; 1.06]	0.065
			0.36 [− 0.23; 0.94]				− 0.31 [− 0.91; 0.30]		73%	
			Small				Small		Large	
FM, kg	25.8 ± 7.1	26.0 ± 7.1	0.17 [− 0.33; 0.67]	1.000	26.7 ± 7.1	26.8 ± 7.1	0.12 [− 0.39; 0.64]	1.000	0.04 [− 0.67; 0.77]	0.900
			0.39 [− 0.20; 0.98]				0.22 [− 0.38; 0.82]		57%	
			Small				Small		Small	
FM, %	35.8 ± 5.5	36.0 ± 5.5	0.19 [− 0.62; 1.00]	1.000	39.1 ± 5.7	39.4 ± 5.7	0.27 [− 0.59; 1.14]	1.000	− 0.08 [− 1.26; 1.11]	0.900
			0.16 [− 0.41; 0.73]				0.32 [− 0.28; 0.93]		51%	
			Trivial				Small		Trivial	

Data are estimated marginal means ± standard deviation. a Cohen’s dz are corrected by Hedges g to account for small sample sizes.

*95% CI* confidence interval at 95%, *AEG-CG* stochastic superiority of interaction of the time (post- vs. preintervention) and group (EG vs. CG), *FM* fat mass, *MD* marginal mean difference, *MM* muscle mass, *Pre-* preintervention assessment, *Post-* postintervention assessment, *WC* waist circumference.

Significance: *p < 0.05, **p < 0.01, ***p < 0.001.

### Hemodynamic changes

Overall, there were no significant interaction effect or group differences (EG vs. CG) in resting heart rate (HRrest), systolic blood pressure at rest (SBPrest), diastolic blood pressure at rest (DBPrest) or mean resting blood pressure (MBPrest). However, there was an effect of time, revealing that at postmeasurement, SBPrest reduced by 6.5 mmHg (95% CI [−12.2; −0.8], *t*_20_ = −2.22, *p* = 0.038). Moreover, fixed effects estimated parameters showed a postintervention effect of sex, indicating that males had lower values than females for SBPrest (β = −15 mmHg, 95% CI [−26.7; −3.3], *t*_20_ = −2.51, *p* = 0.021), DBPrest (β = −8.4 mmHg, 95% CI [−14.8; −1.9], *t*_20_ = 2.53, *p* = 0.020), and MBPrest (β = −10.6 mmHg, 95% CI [−18.3; −2.9], *t*_*20*_ = -2.68, *p* = 0.014). Later, simple effects analysis revealed that the intervention was especially effective for males of the EG since they reduced their SBPrest (β = −18.0 mmHg, 95% CI [−30.9; −5.11], *t*_20_ = −2.91, *p* = 0.009) and MBPrest (β = −12.7 mmHg, 95% CI [−23.4; −1.9], *t*_20_ = −2.45, *p* = 0.023). The random intercepts were moderate for HRrest (ICC = 0.692) and SBPrest (ICC = 0.613) and low for DBPrest (ICC = 0.271).

### Functional performance changes

**Figure 1:**
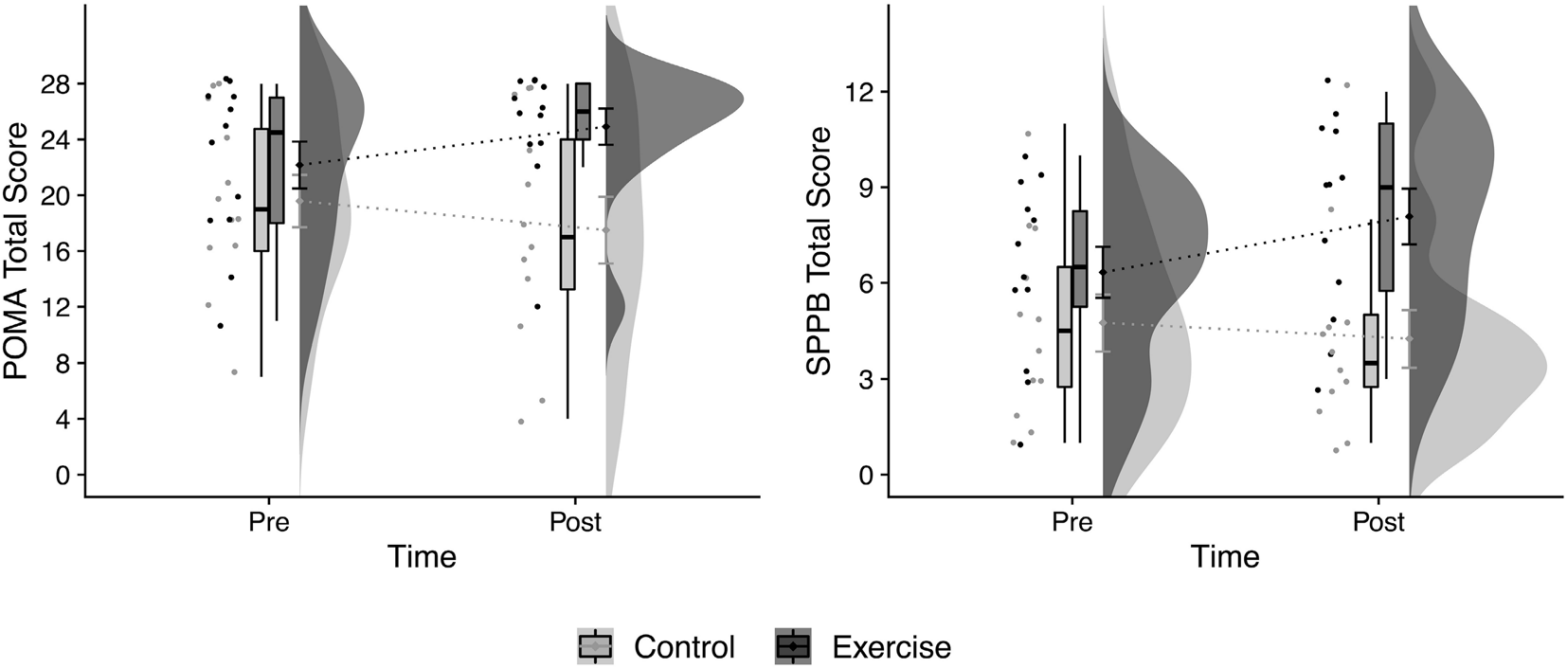
Raincloud plot^46^ of the changes in the Performance-Oriented Mobility Assessment total score (POMA-T) and the Short Physical Performance Battery total score (SPPB-T) in the exercise (n = 12) and control groups (n = 12). Points are raw data obtained for each participant at each moment of measurement. Box plots represent the median (i.e., the line near the middle of the box) and interquartile range (IQR, lower quartile = 25th percentile and upper quartile = 75th percentile). The whiskers on either side of the IQR represent the lowest and highest quartiles of the data. The ends of the whiskers represent the maximum and minimum data values, and the individual dots beyond the whiskers represent outliers in the data set. Clouds represent probability density, and point and error bars in the base of the clouds are the mean ± SD. The dotted line represents the direction of the pre- and postintervention change in the two group means.

Functional changes evaluated throughout the qualitative functional test (Performance-Oriented Mobility Assessment total score, POMA-T) showed a significant effect of the intervention (*F*_1,∞_ = 23.86, *p* < 0.001) (Table 3). The EG presented a 96% likelihood of achieving greater changes, improving POMA-T scores, than did the CG (see Fig. 1). Similarly, there was a significant interaction (time × group) on the POMA balance, POMA-B (*F*_1,∞_ = 12.15, *p* < 0.001) and POMA gait, POMA-G (*F*_1,∞_ = 19.70, *p* < 0.001) with larger likelihoods of higher values for the EG (AEG-CG = 83% and 94%, respectively) than for the CG. Post hoc analyses revealed only significant changes in the EG, specifically on the POMA-T (*p* = 0.016) and POMA-G (*p* = 0.008), but not on the POMA-B (*p* = 0.156). Functional changes evaluated throughout the quantitative functional test (Short Physical Performance Battery total score, SPPB-T) showed a significant effect (*F*_1,∞_ = 23.77, *p* < 0.001) of the intervention (Table 3). The EG presented a 93% likelihood of achieving greater changes in scores on the SPPB-T than did the CG. Furthermore, we found an interaction (time × group) on the SPPB gait speed, SPPB-G (*F*_1,∞_ = 11.75, *p* = 0.001, AEG-CG = 81%) and on the SPPB balance, SPPB-B (*F*_1,∞_ = 6.67, *p* = 0.010, AEG-CG = 72%); nevertheless, an SPPB chair-stand (SPPB-ChS) interaction (*F*_1,∞_ = 1.81, *p* = 0.179, AEG-CG = 66%) was not significant. Post hoc comparisons revealed no significant changes in SPPB components and total scores in the CG. Notwithstanding these comparisons, the EG moderately improved the SPPB total score [*p* = 0.016, stochastic superiority of post- vs. preintervention (Apost-pre) = 67%], showed no significant improvement on the SPPB-G (*p* = 0.080, Apost-pre = 67%), and showed no significant changes on the SPPB-B (*p* = 0.408, Apost-pre = 59%) and the SPPB-ChS (*p* = 0.152, Apost-pre = 59%).

**Table 3:** Changes in functional performance evaluated throughout the performance-oriented mobility assessment (POMA) and short physical performance battery (SPPB).

	EG		Apost-pre	*p* value	CG		Apost-pre	*p* value	*p* value time x group
	Pre-	Post-			Pre-	Post-			A_EG-CG_
**POMA** total score (max 28 points)	24.5 [18.0; 27.0]	26.0 [24.0; 28.0]	64% medium	0.016*	19.0 [16.0; 24.8]	17.0 [13.3; 24.0]	42% small	0.068	< 0.001***
									96% large
POMA balance (max 16 points)	15.5 [10.8; 16.0]	16.0 [15.8; 16.0]	64% medium	0.156	12.0 [9.8; 16.0]	11.0 [7.8; 15.3]	40% small	0.168	< 0.001***
									83% large
POMA gait (max 12 points)	9.0 [7.8; 11.0]	10.0 [9.0; 12.0]	63% small	0.008**	7.5 [4.8; 10.3]	7.5 [3.8; 10.3]	45% small	0.436	< 0.001***
									94% large
**SPPB** total score (max 12 points)	6.5 [5.3; 8.3]	9.0 [5.8; 11.0]	67% medium	0.016*	4.5 [2.8; 6.5]	3.5 [2.8; 5.0]	44% small	0.524	< 0.001***
									93% large
SPPB balance (max 4 points)	3.0 [1.8; 4.0]	3.0 [3.0; 4.0]	59% small	0.408	2.0 [1.8; 3.0]	2.0 [1.0; 2.0]	42% small	0.408	0.009**
									72% large
SPPB gait speed (max 4 points)	3.0 [1.0; 3.0]	3.0 [1.8; 4.0]	67% medium	0.080	1.0 [1.0; 2.3]	1.0 [1.0; 1.3]	42% small	0.332	0.001**
									81% large
SPPB chair-stand (max 4 points)	1.5 [1.0; 2.0]	1.5 [1.0; 3.3]	59% small	0.152	1.0 [0.0; 1.0]	1.0 [0.0; 1.3]	52% small	1.000	0.179
									66% medium

Data are the median [Q25; Q75].

*AEG-CG* stochastic superiority of the interaction of the time (post- vs. pre-) and the group (EG vs. CG), *Apost-pre* stochastic superiority of post- versus pre-, *Pre-* preintervention assessment, *Post-* postintervention assessment.

Significance: *p < 0.05, **p < 0.01, ***p < 0.001.

Individual responses showed a clear evolution of EG patients toward higher scores on the POMA-T and SPPB-T, reaching a “ceiling effect” on the POMA-T at postintervention (Fig. 1).

### Correlations

**Figure 2:**
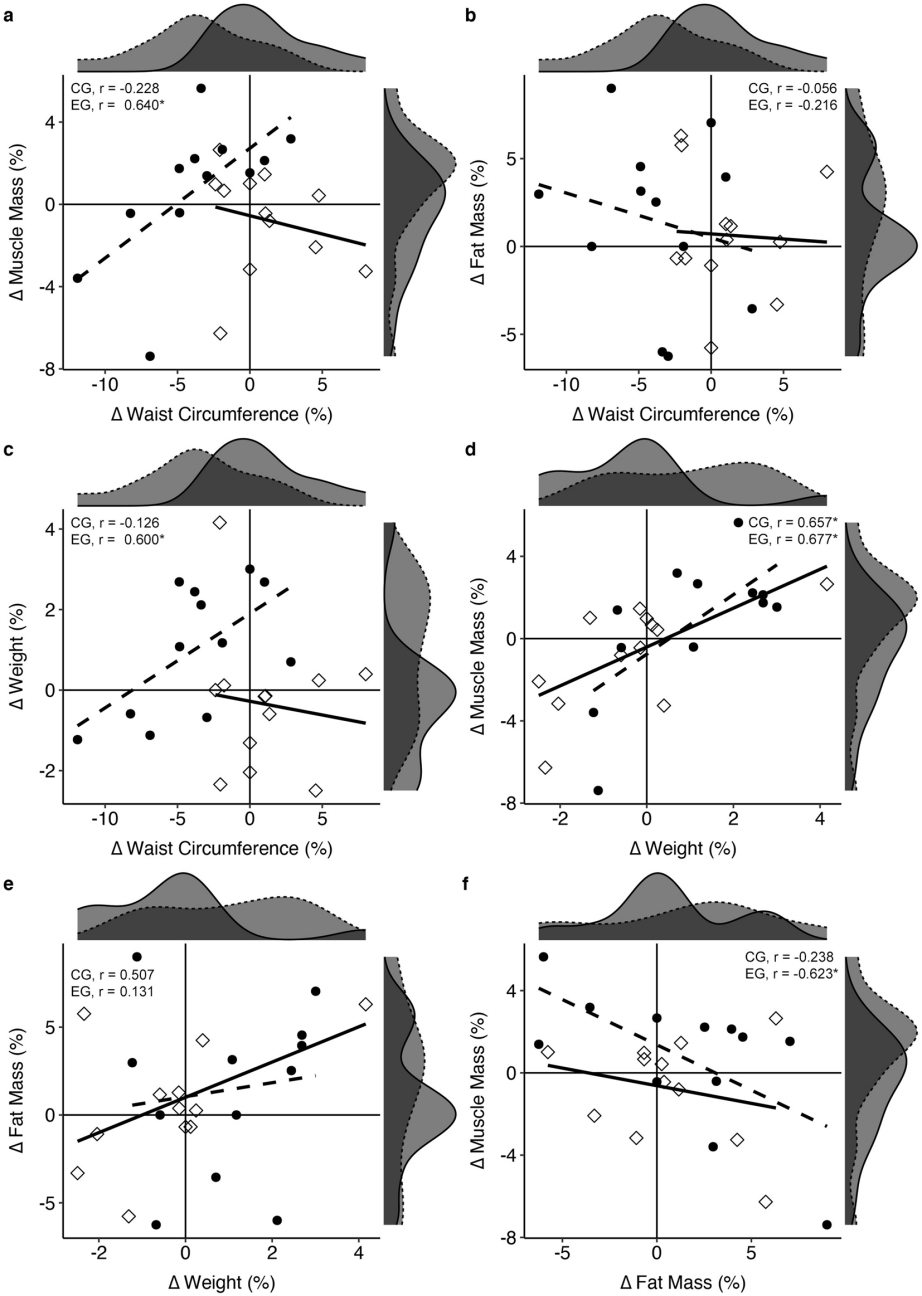
Pearson correlations between percentage changes in body composition variables. Changes in the control group (CG) are represented by a solid line, diamond points, and solid line density plot. Changes in the exercise group (EG) are represented by a long-dashed line, filled circle points, and a dashed line density plot. Significance: *p < 0.05, **p < 0.01, ***p < 0.001.

There were no significant associations between changes in body composition, hemodynamic parameters, and functional performance variables. In the EG, a positive association was observed between the percentage of change in WC (∆ WC, %) and the percentage of change in muscle mass (∆ MM, %) (*r* = 0.640, *p* = 0.025). As seen in Fig. 2 (section a), this association was drawn by a reduction in WC and an increase in MM (see dashed line density curves). No association was found between WC and change in fat mass, ∆ FM (Fig. 2, section b). A positive association was also observed between WC (∆ WC, %) and weight (∆ weight, %) (*r* = 0.600, *p* = 0.039), with a reduction in WC and an increase in weight (see Fig. 2, section c). Furthermore, ∆ MM (%) was positively associated with ∆ weight (%) (*r* = 0.677, *p* = 0.016, see Fig. 2, section d) and negatively associated with ∆ FM (%) (*r* = −0.623, *p* = 0.031) (see Fig. 2, section f). On the other hand, in the CG, a positive association was only observed between ∆ weight (%) and ∆ MM (%) (*r* = 0.657, *p* = 0.028) (see Fig. 2, section d), but no association was found between ∆ FM and ∆ MM (Fig. 2, section e).

## Discussion

Short-term exercise training (i.e., 6 weeks) consisting of a lower volume of low-to-moderate intensity cycling training with simultaneous upper and lower limbs significantly reduced waist circumference and improved functional performance, especially motor skills related to balance and gait, in institutionalized older adults with multimorbidity.

To the best of the authors’ knowledge, there is a lack of studies that have applied low-to-moderate intensity-controlled exercise training in older adults. We found evidence of an association between WC reduction and fat mass (skinfold) loss with light-to-moderate (high-light) PA in older adults^23,24^. Previous cross-sectional studies have indicated that higher volumes of PA (>300 min/week) than the exercise dose employed in our intervention (60 min/week) are necessary to observe higher body composition changes. However, the effect of weekly cumulative training cannot be observed in these studies, and yet in our study, the participants completed a total of 18 cycling sessions of 20 min of progressively increased intensity. Weight estimation increased up to 1.29 kg (*p* = 0.007), and muscle mass increased up to 530 gr (*p* = 0.065) in the EG compared to the CG. Furthermore, we were 73% (large) more likely to find one participant in the EG who had increased weight than in the CG, and we had the same likelihood of finding one participant in the EG who had augmented muscle mass than in the CG. In fact, the association analysis showed a strong significant relationship in the EG between changes in body weight and changes in muscle mass and no significant relationship with respect to changes in fat mass. As observed in Fig. 2, section d, two-thirds of participants in EG increased their muscle mass; therefore, a similar positive relationship between changes in muscle mass and changes in weight in both groups has a different interpretation. Participants in the EG gained weight that was partially attributable (i.e., 43%) to the increase in muscle mass, and approximately half of the participants in the CG showed a reduction in weight partially attributable to a reduction in muscle mass. Furthermore, percent changes in muscle mass in the EG were strongly related to the percentage reduction in waist circumference. On the other hand, the CG mainly showed stabilization or a reduction in weight with no changes or reductions in muscle mass. Importantly, these results indicate that participants in the EG augmented their weight with a concomitant muscle mass gain and a reduction in abdominal fat. On the other hand, the CG mainly showed stabilization or a reduction in weight with no changes or reductions in muscle mass. The low physical condition of the participants and the increase in cycling intensity in the final three-week period of the training intervention likely contributed to the body composition changes in the EG. Notably, half of the participants in the CG showed muscle mass reductions in only 6 weeks, even though they were performing their habitual activities. These findings highlight the need to implement specific and individualized exercise interventions in older adults to slow down or even reverse age-related muscle mass reduction, which can be related to sarcopenia. When adjustments were made for sex, the single-effect analysis confirmed the increase in muscle mass in men. It can be hypothesized that the sex dependence observed for muscle mass changes may be due to less hypertrophy capacity in menopausal older women or to the effect of any pathology, specific diet, or medicines to treat it on muscular protein synthesis^25^.

We observed a time effect without group differences in resting heart rate and blood pressure. However, simple effects analysis showed that males from the EG had reduced SBP (∼15 mmHg) and MBP (∼13 mmHg) after the intervention. It has been highlighted that dynamic endurance training consistently reduces blood pressure^26^, even matching the effects of first-line medications^27^. Participants from our study highly reduced their SBP by more than 8.96 mmHg, a value similar to that reported in a recent systematic review with meta-analysis, where 391 randomized controlled trials were analyzed^27^. In the aforementioned review, it was already found that being male, being older than age 50, and having been diagnosed with hypertension were factors that favored chronic reductions in BP after dynamic endurance training^26^. There is a debate in the literature about the exercise dose that should be recommended to optimize the chronic hypotensive effect. Cornelissen and Smart^26^ signaled that thirty to forty-five min of a moderate-to-high intensity dose maximizes BP reductions. Nevertheless, in the present study, a great reduction in SBP in older men with multimorbidity after a lower dose of exercise, specifically 60 min per week of simultaneous limb cycling training, was found. Increasing data suggest that there are sex differences in ventricular and vascular adaptations to aerobic (endurance) exercise that evoke a differential balance of regulatory challenges for the cardiovascular system of aging women compared to men^28^. The sex differences during exercise, after exercise, and in response to exercise training are not completely known, and our study does not have a sufficient sample size to address this issue. Additionally, the effect of medications on BP and their interaction with the exercise intervention could have influenced the different responses between males and females in our study^27^. Future studies should elucidate the effect of different doses of aerobic exercise in older adults and their interactions with sex, pathologies, and medicines.

Overall, low-to-moderate cycling training improved functional performance, reflected by the total scores on the POMA and SPPB. Balance and gait were the motor skills that benefited most; in contrast, there was no improvement in the chair-to-stand test. The performance changes observed were consistent with the specificity of the adaptations to exercise training^29^. Gait and balance require interlimb coordination and less muscle tension to be applied on bone levers; however, standing from a chair requires interlimb coordination with a higher level of muscle tension. In our exercise intervention, we opted to include a sample of older adults with multimorbidity and low functional mobility and potentially increased risk of death according to the interquartile range of their SPPB total scores (Q_25_ = 5.3 points and Q_75_ = 8.3 points)^30^ through involving them in cycling training. Our exercise design has proven to be beneficial and safe to improve the functional performance of frail older adults; however, multicomponent training should be considered to improve the capacity to rise from a chair, which implies higher levels of strength than walking or maintaining a static posture^31^. Cycling is classified within endurance types of exercise in the continuum strength-endurance exercise; however, when employing a cycling device, a valuable strategy would be the increase in resistance to the pedal to contribute to a more strength-oriented training, facilitating strength gains and becoming a first step before the introduction of weight-bearing exercise. In the present study, the EG improved their SPPB-T scores by 2.5 points, which is more than double what is considered a substantial change (i.e., definitely important change = 1 point^30^).

Notwithstanding the abovementioned results, participants in the CG substantially decreased their SPPB scores. On the other hand, the POMA increased by 2 points in the EG and decreased by 2 points in the CG, which highlights a greater improvement than the 0.8 points reported as a reliable change in the mean score^32^. It has been suggested that the POMA-T has a ceiling effect that makes it less sensitive to detecting changes in fitter participants; thus, it is recommended to apply it always as part of a battery of tests and not to rely exclusively on that test to study changes after an exercise intervention. Overall, we found that the daily activities performed by this sample of older adults were not enough to maintain functional performance for 6 weeks. The exercise intervention led to 93% and 96% likelihoods that a randomly selected change in functional performance from the EG would be greater than a randomly selected change score from the CG.

Our study has several potential limitations. First, the duration of the study comprised 6 weeks of intervention, and although in that short time, participants improved body composition and functionality with low-volume training and mild-to-moderate intensity, it would be necessary to verify in future studies whether by prolonging the duration of training in longer periods, a progressive improvement would be observed. Identifying a minimum dose of improvement is very important in populations of older adults with comorbidities since the use of high doses of training would not be feasible in a clinical context and could also reduce the users’ adherence. Moreover, future studies should analyze the minimum doses necessary to produce greater benefits in this population. Furthermore, although the control of physiological variables (heart rate, blood pressure, and oxygen saturation) was made three times during the sessions (before, during, and after), the continuous use of cardiac monitors and a gas analyzer could provide valuable information to assess the physiological changes and the adaptation mechanism of the participants along with the session. On the other hand, the intervention used in this study must be complemented with exercises to improve muscle power. In the present study, physical function improved in walking and balance activities, but no improvement in the time spent in the chair-to-stand test was observed. The reduction in force and velocity experienced by older people leads to a critically affected ability to generate muscle power or the ability to perform movements that require a manifestation of force at a certain speed. Two decades ago, muscle power was believed necessary to improve performance in sports activities; however, during the last ten years, it has been known that it is also an essential component in the performance of certain activities of daily living and that is closely linked with improved functionality^33^. An adequate level of neuromuscular power will help older people decelerate their movement to change their spontaneous direction of gait or stop their movement in a situation that poses a certain risk of falling; it will also allow them to rebalance in the face of an external disturbance (e.g., a stone in the road, a boost on the bus, etc.). Indeed, it has been suggested that muscle power is a more discriminating predictor of functional performance in older adults than muscle strength^33,34^. Finally, the present study has a small sample size, and despite that the individual behavior of the participants was analyzed by statistical methods and graphic reporters, future studies should replicate this training program in a larger sample. It is necessary to improve the exercise prescription in older people with comorbidities, and low-cost exercise prescription and monitoring tools are easily implemented in clinical settings, facilitating the practice of exercise as a habit, especially in nursing homes.

## Conclusions

A total of 20 min of cycling training 3 days per week at low-to-moderate intensity improved body composition and increased walking and balance performance in only 6 weeks in older adults with multimorbidity. Furthermore, we found a positive effect on resting blood pressure in men. Further studies should consider studying different adaptations to cardiovascular training in women. Moreover, perceptually regulated intensity during exercise training seemed to be a very interesting strategy to train people with low physical fitness and multimorbidity. The perceptually regulated exercise test (PRET) is inexpensive, safe, easily applicable, and allows for individualized control of the internal load, especially in older people who consume β-blockers. Therefore, it seems reasonable to encourage long-term care settings to introduce exercise programs to promote long-term physical independence and reduce the development of sarcopenia in older adults with multimorbidity. These programs should be dosed with tools that allow for intensity individualization and start with a low dose and gradually increase it. Our study has shown very good outcomes using doses considerably lower than the WHO recommendations for older adults. More studies are warranted to investigate the minimum and optimal doses of exercise that produce health benefits in older adults with multimorbidity.

## Methods

### Study design

The study conducted between September and December 2019 was a 2 × 2 randomized controlled trial using a two-group design (exercise vs. control) and two repeated measures (pre- vs. postintervention). The exercise group was requested to accomplish a low volume (i.e., 20 min, 3 days per week) and low-to-moderate intensity (i.e., 3–6 on the rating of perceived exertion (RPE) scale of 0–10) combining upper and lower limb recumbent cycling training for six weeks. Participants were evaluated the week before and the week after the training period (pre- vs. postintervention) to facilitate an examination of the changes in body composition, functional performance, and resting cardiovascular state. Furthermore, participants were monitored physiologically during each session (HR and blood pressure) to control any possible adverse effects. Both groups were familiarized with the former test procedures, and only the exercise group was familiarized with the cycling exercise and perceived effort scale during the week before the evaluation. This study was performed in accordance with the CONSORT (CONsolidated Standards of Reporting Trials) 2010 guideline.

### Participants

**Figure 3:**
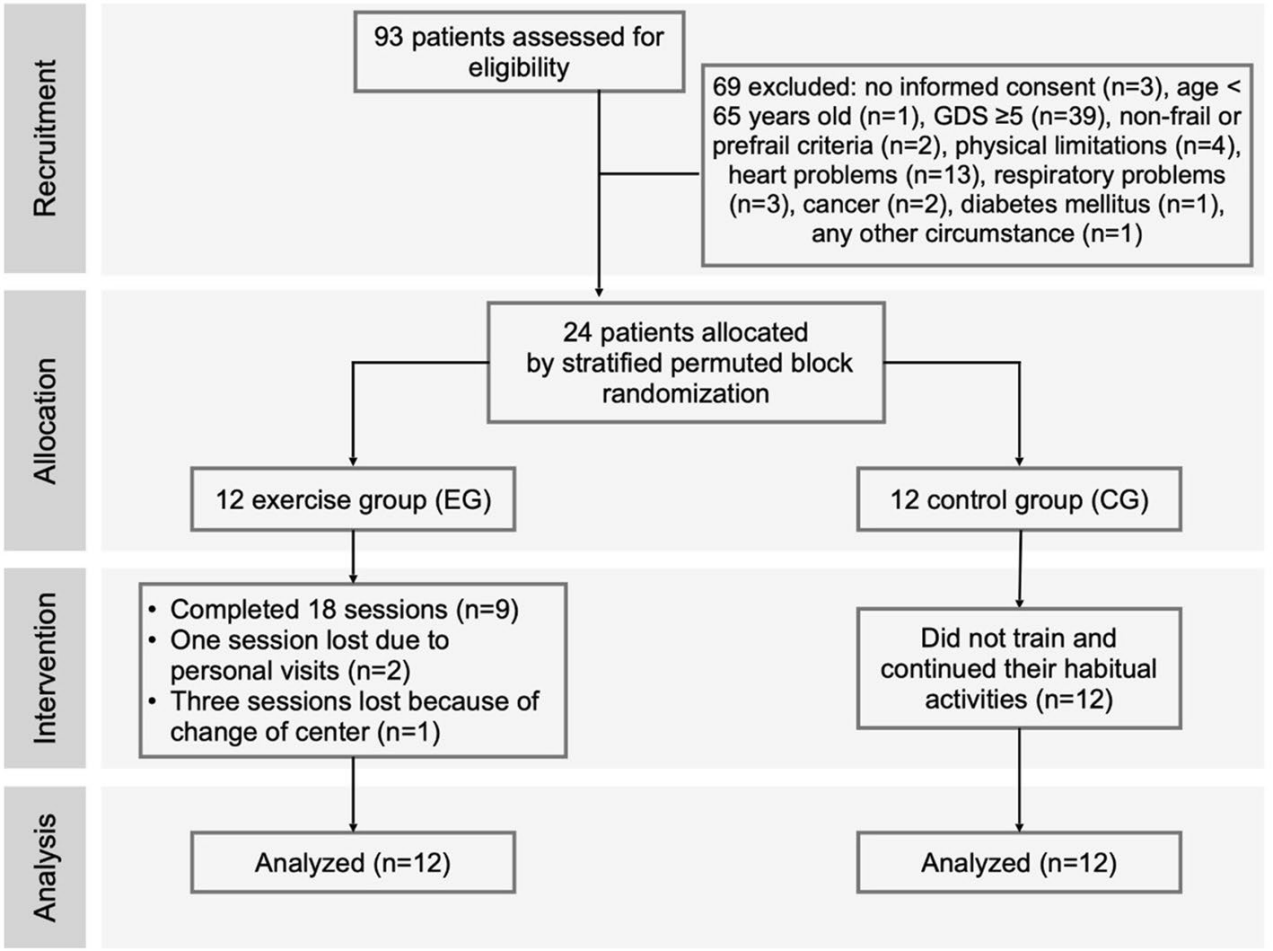
Consort flow chart for the selection and allocation of participants in the exercise and control groups.

Participants were recruited between September and October 2019 at the Gerontological Complex La Milagrosa (A Coruña, Spain), consisting of a daycare center and a nursing home. Core services provided by the daycare center include small-group training on memory, activities of daily living, reality orientation, and cognitive stimulation. Nursing home services additionally offer a 24-h room, supervision, and nursing care (personal care, medication management, administration, palliative care, rehabilitation, activities, and transportation). The multidisciplinary research team established the inclusion and exclusion criteria intending to minimize the possible risks of the intervention. The inclusion criteria were as follows: (a) men and women aged 65 and older, (b) users of a care setting—daycare patients or nursing home residents, and (c) a score < 5 in the Global Deterioration Scale (GDS), from no cognitive decline to moderate cognitive decline^35^. Exclusion criteria were as follows: (a) physical limitations or musculoskeletal injuries that could affect cycling training performance; physical exercise contraindicated by the physiotherapist and verified by the medical doctor according to the medical register of each participant; (b) heart failure with a functional class according to the New York Heart Association (NYHA) Classification of NYHA III and IV^36^; (c) the presence of acute pain that does not allow exercise training; (d) recent acute myocardial infarction (in last 6 months) or unstable angina; (e) uncontrolled hypotension; (f) uncontrolled arterial hypertension (>180/100 mmHg); (g) active cancer treatment with chemotherapy; (h) patients with an active pacemaker and/or uncontrolled block; (i) diabetes mellitus with acute decompensation or uncontrolled hypoglycemia; or (j) any other circumstance that precludes individuals from completing the training intervention. After the application of the inclusion and exclusion criteria, a total of 24 participants were recruited and randomly placed into two groups: the exercise group (EG, n = 12) and the control group (CG, n = 12). Both groups continued with the daily routines of the center and maintained diet and medication patterns throughout the entire study. A stratified permuted block randomization was employed that accounted for the GDS score, sex, and type of institutionalization (Fig. 3). A nurse and a medical doctor enrolled and assigned participants to intervention.

All subjects were informed in advance about the study and gave their written informed consent to participate in the study, either directly or through their legal representatives. The present study was carried out following the process approved by the Autonomic Research Ethics of Galicia Committee, Spain (code 2018/010), and in agreement with the Declaration of Helsinki. The study was registered with ClinicalTrials.gov (NCT04842396). Registered 13/04/2021-Retrospectively registered.

### Measures

#### Inclusion phase

Demographic (patient age and sex) and clinical [weight, height, body mass index (BMI), use of a walking aid, GDS score, morbidity conditions, and medication] characteristics of the participants were collected during the inclusion phase. Weight and height were measured according to standardized protocols (calibrated digital scale and stadiometer, respectively), and BMI was calculated by dividing weight (kilograms) by the square of height (in meters). The Charlson Comorbidity Index adjusted by age (CCIage)^37^ was calculated as a prognostic index of comorbid conditions for patients allocated in both groups of the present study. Nine of the 19 Charlson comorbidity conditions and the presence of hypertension diagnosis were also included. The total number of medicines consumed and information on antihypertensive drugs such as beta-blockers and other drugs to lower blood pressure were collected. All medical information was registered from the individual’s medical history.

#### Pre-postevaluation

The week before and the week after six weeks of simultaneous cycling training, both groups completed the assessment protocol for evaluating the impact of the intervention. This assessment protocol consisted of the evaluation of body composition, hemodynamic parameters, and functional performance. The entire evaluation process was carried out in days, keeping the same order in the pre- and postintervention.

##### Body composition evaluation

The body composition was measured by bioimpedance analysis according to the instructions of the manufacturer (Inbody 270; InBody USA, Cerritos, CA, USA). Several parameters, such as body weight, muscle mass (MM), fat mass (FM), and FM percentage, were automatically measured. Waist circumference (WC, cm) was taken at end tidal using a measuring tape to the nearest 0.1 cm, midway between the lowest rib and the iliac crest, which corresponded with the level of the umbilicus. Repeat measurements were made, and the mean value of two measures, which showed agreement within 0.5 cm, was used.

##### Hemodynamic parameter evaluation

After body composition evaluation and matching at approximately 3 h postprandial, a trained nurse evaluated hemodynamic parameters. Participants were not allowed to consume alcohol or caffeine during the 24-h period preceding the measurement day, and they took their medications at least one hour before the measurements. Patients sat comfortably for a minimum of 5 min before the measurement with their backs resting on a chair and avoiding crossing their arms and legs. The baseline hemodynamic state was characterized by storing the mean of the three lowest values for thirty seconds of heart rate (HRrest) with a finger pulse oximeter (PO40, Beurer Medical GmbH, Ulm, Germany). A well-trained physician determined blood pressure by the auscultator method using a properly calibrated mercury column sphygmomanometer flexible cuff of the appropriate size (Tycos Classic Hand Aneroid, 5098-02, Welch Allyn Inc.) and a stethoscope (Littmann, Classic III, United States). At least three systolic (SBPrest) and diastolic blood pressure (DBPrest) measurements were recorded at 1-minute intervals. Mean blood pressure (MBPrest) was calculated as follows:

$$ MBP = DBP + \frac{1}{3}{\text{~}}\left( {SBP - DBP} \right) $$

##### Functional evaluation

Both groups were tested employing functional test batteries that have been extensively employed in the literature with good to excellent values of inter- and intraclass reliability^30,31^. On the first day of evaluation, and in conjunction with body composition and hemodynamic assessment, participants completed the Performance-Oriented Mobility Assessment (i.e., POMA^38^), which measures balance (i.e., POMA-B, nine maneuvers graded as either normal, adaptive, or not normal and scored up to 16 points) and gait performance (i.e., POMA-G, seven maneuvers graded as normal or abnormal and scored up to 12 points) used during normal daily activities evaluated by observation and graded on an ordinal scale where participants can obtain up to 28 points in the total score (i.e., POMA-T) depending on the quality of movement. The Short Physical Performance Battery test (i.e., SPPB^39^) was employed to evaluate the time spent to complete three balance tasks (i.e., SPPB-B), walk 4 meters at a comfortable speed (i.e., SPPB-G), and sit-to-stand 5 times from a chair (i.e., SPPB-ChS). This test battery provides a summary score ranging from 0 (worst performers) to 12 (best performers). The SPPB test is a valid instrument for screening frailty and predicting disability, institutionalization, and mortality. A total score of less than 10 indicates frailty and a high risk of disability and falls, and a 1-point change in the score has clinical relevance^30^. Walking aids were allowed for the SPPB; however, if they were used, the same conditions were employed in the postevaluation.

### Familiarization

Participants familiarized themselves with the activities over 2 days of 4–16 min of simultaneous upper and lower limb cycling at a comfortable pace, progressing until volitional stop. Older adults were seated in a geriatric chair that was adapted to allow a knee joint range of movement of approximately 42° (i.e., knee extension 152° and knee flexion 110°) and cycled on a recumbent motorized cycle (MOTOmed muvi Movement Therapy Trainer; Reck-Technik GmbH & Co., Betzenweiler, Germany). The MOTOmed muvi has a display where velocity (m/s) and resistance (kg) can be observed when cycling; thus, within the familiarization session, researchers annotated the velocities employed for the participants cycling 2 min with 1 kg and encouraged them to maintain this cadence when the load was increased at a rate of 2 kg each 2-min stage. The trial was terminated when the participant volitionally stopped exercise owing to fatigue or discomfort. Furthermore, the trial was finished when the investigator determined that the participant could not maintain the designated pedaling rate for more than 10 consecutive seconds at the current load.

Before they began cycling, participants were anchored to the perception scale using a combination of exercise and memory procedures. This procedure required the participant to cognitively establish a perceived intensity of effort that is consonant with that depicted visually by a cyclist figure at the bottom (i.e., low anchor, rating 0) and top (i.e., high anchor, rating 10) of the hill, as presented in the OMNI-RPE scale illustrations^40^. The OMNI-RPE scale was visible at all times of the progressively increased resistance cycling exercise, and participants indicated a number before starting cycling and in the last 15 s of each stage. The familiarization period was conceived to train participants to anchor a score on the OMNI-RPE against a spectrum of intensities, defined by resistance applied, ensuring that they stop voluntarily at least once due to an inability to maintain the cadence set or a symptom-related alert (i.e., leg tightness/pain, dizziness, chest tightness/pain) that prevented them from exercising safely.

### Training period

The exercise group cycled 20 min per session on the MOTOmed muvi 3 days per week for 6 weeks at an intensity guided by the perception of effort. Participants were seated in a geriatric chair that was adapted to the measures taken during familiarization, and a knee joint range of movement of approximately 42° was adopted.

All sessions on the motorized cycle ergometer were completed between 10:20 am and 1:30 pm to avoid possible chronobiological effects on training adaptation.

A cycling cadence was fixed between 25 and 30 rpm for all sessions since that cadence was comfortable for every participant. Researchers adjusted resistance on the motorized cycle to increase the external load until it reached the level required to reach the intensity of effort programmed by the OMNI-RPE. The 6 weeks were programmed in the form of two intensity-differentiated training phases of three weeks. In the first training phase (i.e., the first three weeks), participants were requested to cycle simultaneously with the upper and lower limbs at an intensity equivalent to a perception of 3 (i.e., easy to somewhat moderate) on the OMNI-RPE (0–10). Subsequently, in the second training phase (i.e., the last three weeks), they were requested to cycle at an intensity equivalent to an OMNI-RPE score of 6 (i.e., somewhat hard). The participants cycled a mean load per session of 18.2 (SD −2.9) watts with their arms and 35.5 (SD −4.7) watts with their legs in the first training phase and 25.2 (SD −6.8) watts with their arms and 41.8 (SD −8.3) watts with their legs in the second training phase. To regulate effort according to the programmed intensity, the researchers frequently requested that the participants anchor a score on the OMNI-RPE scale, asking, “How effortful is the task?” Moreover, control of adverse events throughout the trial was measured through the assessment and monitoring of vital signs before, during (within the first 10 min), and after the intervention sessions. Vital signs [heart rate (per minute), systolic and diastolic blood pressure (in millimeters of mercury, mm Hg), and oxygen saturation (in percentage)] were monitored by a nurse and a medical doctor using mobile finger pulse oximeters (Riester, Germany). Professionals identified whether patients’ rates were within the normal range for each vital sign. The internal load was monitored and adjusted in case the observed values were outside of the normal range. During the clinical trial, only one woman (in two sessions) had to stop the activity due to the high increase in her heart rate and had to perform the session on the next day. In addition to these measures, the medical doctor performed a visual control assessment of the participants and asked them whether they felt comfortable with the exercise.

### Data analysis and statistics

Data are presented as the median and interquartile range [Q25; Q75] for ordinal variables (i.e., scores) and the estimated marginal mean ± standard deviation (SD) for continuous variables. Normality was assessed through standard distribution measures, visual inspection of Q–Q plots and box plots, and the Shapiro–Wilk test. The effect of the intervention on the ordinal variables of the POMA and SPPB was analyzed employing nparLD (nonparametric analysis of longitudinal data in factorial experiments) from the R software package^41^. This package is a nonparametric ANOVA-type statistic (group × time) that uses ranks for calculating relative marginal effects, and it was chosen because unlike traditional nonparametric tests, this test provides the effect of each factor and the interaction between them. When a significant interaction was detected, comparisons were applied using the Wilcoxon signed-rank test and the Mann–Whitney U test for paired within-group and independent between-group variables, respectively. Multiple sequentially rejective Bonferroni-Holm tests were employed for the correction of multiple comparisons. Changes within and between groups in body composition and hemodynamic parameters were analyzed by employing mixed models for repeated measures designs with the module GAMLj, which uses the R formulation of random effects as implemented by the lme4 R package in jamovi software^42^. GAMLj estimates variance components with restricted (residual) maximum likelihood (REML), which, unlike earlier maximum likelihood estimation, produces unbiased estimates of variance and covariance parameters. The intersubject factor group (EG and CG), the intrasubject factor time (pre- and postintervention), and the interaction (group × time) were set as fixed effects, and participants’ intercepts were set as a random effect. The influence of sex, type of institutionalization, and diagnosed hypertension were introduced as fixed effects only if any of these variables improved the model, as evaluated by the Akaike information criterion (AIC). When a significant interaction was detected, paired and independent comparisons were made with a *t*-test with the Bonferroni-Holm correction for within-subject and between-group changes, respectively. F and *t* values and the corresponding degrees of freedom were computed.

Within-subject changes were evaluated by raw data, mean differences, and standardized mean differences with 95% confidence intervals for continuous variables. The standardized mean difference was calculated as the mean change score divided by the SD of the change score, termed Cohen's dz^43^, and corrected by Hedges’ g to account for small sample sizes. Cohen's dz effect was qualitatively interpreted as trivial if dz < 0.20, small if 0.20 ≤ dz < 0.50, medium if 0.50 ≤ dz < 0.80, large if 0.80 ≤ dz < 1.30, and very large if dz ≥ 1.30^44^. Within-subject changes in ordinal variables were analyzed throughout the stochastic superiority (Apost-pre), which represents the probability that a randomly selected score from the postintervention will be greater than a randomly selected score from the preintervention. Probability values equal to or higher than 0.56, 0.64, and 0.71 when approaching 1 or values equal to or lower than 0.44, 0.36, and 0.29 when approaching 0 for Apost-pre were regarded as small, medium, and large values^45^. Between-group changes were evaluated by the estimated parameter with the 95% CI of the interaction between the fixed effect of the model and by an estimation of the degree of overlap between the changes of two groups throughout the stochastic superiority (AEG-CG) effect size^45^.

Ordinal variables were represented graphically with raincloud plots^46^. These plots allow for the visualization of raw data, probability density, and key summary statistics such as medians, means, and relevant confidence intervals in an appealing and flexible format with minimal redundancy. Associations between changes in body composition and hemodynamic and functional performance tests were analyzed through Pearson or Spearman correlations for parametric and nonparametric variables, respectively. The strength of associations was established as weak, moderate, strong, and very strong according to the cut-off points 0.10, 0.30, 0.50, and 0.70, respectively^44^. The alpha level was *p* < 0.05.
